# Crystal structure of bis­(acetyl­acetonato-κ^2^ *O*,*O*′)(tetra­hydro­furan-κ*O*)(tri­fluoro­methane­sulfonato-κ*O*)iron(III)

**DOI:** 10.1107/S2056989015016849

**Published:** 2015-09-12

**Authors:** Casseday P. Richers, Jeffery A. Bertke, Thomas B. Rauchfuss

**Affiliations:** a School of Chemical Sciences, University of Illinois at Urbana-Champaign, Urbana, Illinois 61801, USA

**Keywords:** crystal structure, iron(III), acac, triflate, tetra­hydro­furan

## Abstract

The mononuclear title complex, [Fe(CF_3_O_3_S)(C_5_H_7_O_2_)_2_(C_4_H_8_O)] or [Fe(acac)_2_(OTf)(THF)] (acac = acetyl­acetonate; OTf = tri­fluoro­methane­sulfon­ate; THF = tetrahydrofuran), (I), consists of one six-coordinate Fe^3+^ atom in a slightly distorted octa­hedral environment [Fe—O bond-length range = 1.9517 (11)–2.0781 (11) Å]. The triflate ligand was found to be disordered over two sets of sites, with a site-occupancy ratio of 0.622 (16):0.378 (16). Weak inter­molecular C—H⋯O and C—H⋯F hydrogen-bonding inter­actions generate a two-dimensional supra­molecular structure lying parallel to (100). This is only the second crystal structure reported of a mononuclear bis­(acetyl­acetonato)iron(III) complex.

## Abstract 2

The mononuclear complex [Fe(acac)_2_(OTf)(THF)] (acac = acetyl­acetonate; OTf = tri­fluoro­methane­sulfonate) consists of a mononuclear six-coordinate Fe^3+^ center in a slightly distorted octa­hedral environment and is only the second crystal structure reported of a mononuclear bis­(acetyl­acetonato)iron(III) complex.

## Chemical context   

Because of its ease-of-handling, relative stability and good solubility in most organic solvents, tris­(acetyl­acetonato)iron(III) [Fe(acac)_3_] is often used as a catalyst or catalyst precursor in iron-catalysed reactions (Sherry & Fürstner, 2008 ▸; Zettler *et al.*, 2001 ▸). In many applications, the loss or substitution of one or more acetyl­acetonate ligands from [Fe(acac)_3_] is expected. However, the substitution of a single acetylacetonato ligand is rarely observed. Relevant examples include protonations of Fe(acac)_3_ with oxalic acid (Fujino *et al.*, 2004 ▸) and hydro­chloric acid (Lindley & Smith, 1970 ▸) to form [Fe(acac)_2_]_2_(μ-C_2_O_4_) and [Fe(acac)_2_Cl], respectively. The dinuclear alkoxides, [Fe(acac)_2_(μ-O*R*)] are also known (Chiari *et al.*, 1984 ▸; Leluk *et al.*, 1992 ▸; Wu *et al.*, 1972 ▸). We now report that the addition of triflic acid to a THF solution of [Fe(acac)_3_] results in the formation of a mononuclear bis(acetyl­acetonato)iron(III) complex, [Fe(acac)_2_(OTf)(THF)], the title compound (I) whose structure is reported herein. This compound is a rare bis­(acetyl­acetonato)iron(III) complex that has been crystallographically characterized.
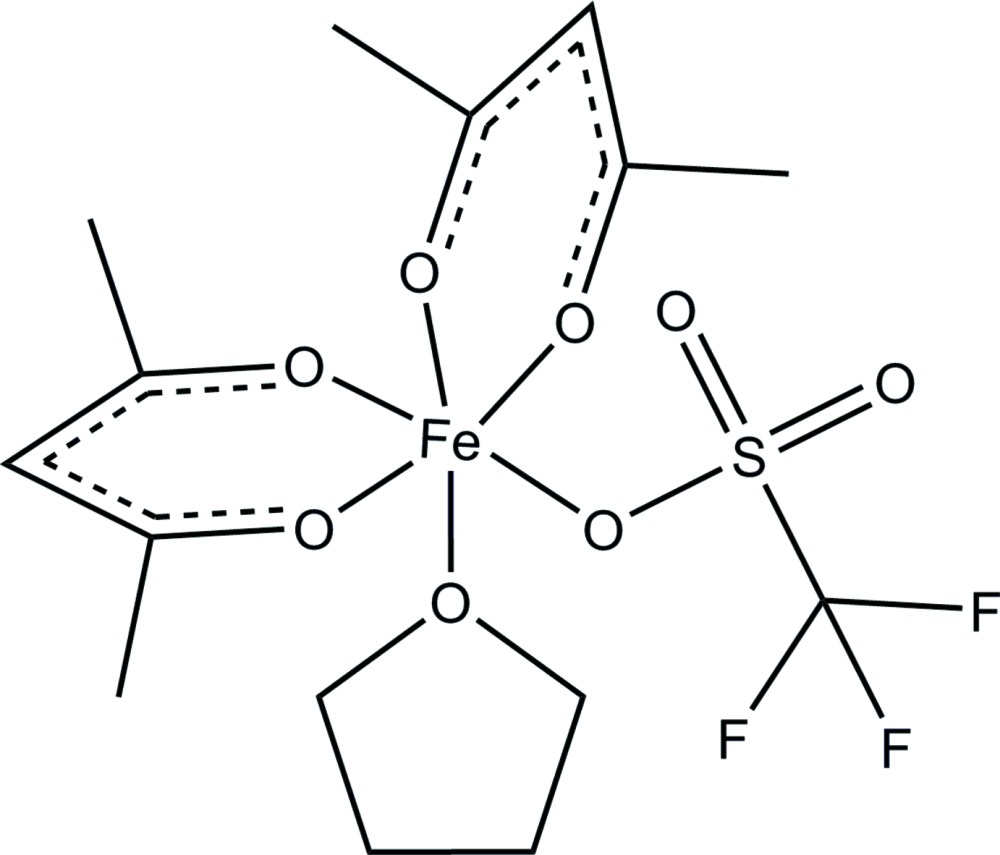


## Structural commentary   

**Figure 1:**
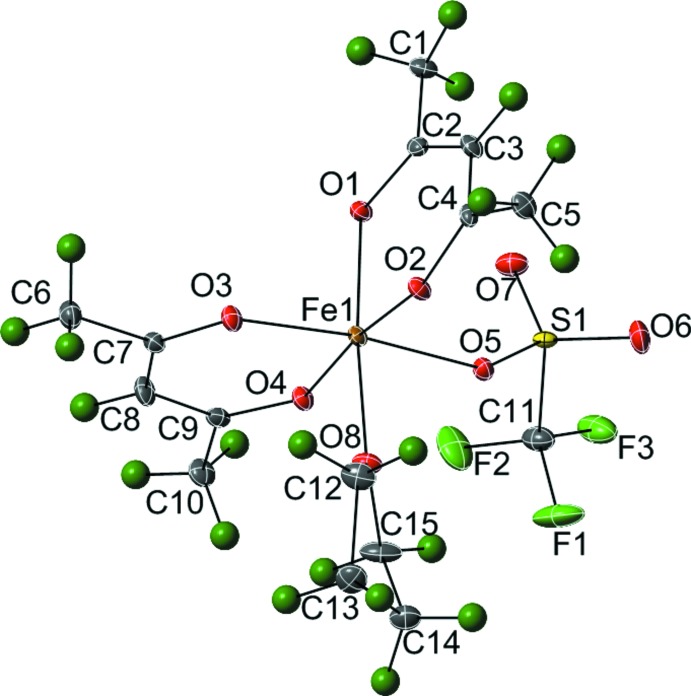
A mol­ecule plot showing the atom numbering, with 35% probability ellipsoids for non-H atoms and spheres of arbitrary size for H atoms. Only the major component of the disordered triflate ligand is shown.

The mol­ecular structure of the mononuclear complex (I) (Fig. 1 ▸) consists of one six-coordinate Fe^3+^ atom in an slightly distorted octa­hedral FeO_6_ environment. The coordination sphere of the metal comprises four oxygen atoms from two κ^2^-acac ligands [Fe—O_acac_ range = 1.9517 (11)–1.9762 (11) Å], one oxygen atom of a THF solvate mol­ecule [Fe1—O8 = 2.0781 (11) Å] and one oxygen atom of a disordered triflate anion [Fe1—O5 = 2.063 (4) Å or Fe1—O5*B* = 2.066 (6) Å] (Table 1 ▸). The disorder in the triflate ligand was found to be 0.622 (16):0.378 (16). The angles around Fe1 deviate from the ideal octa­hedral angles of 90 and 180°, the *cis* angles range from 84.63 (5)° to 98.09 (5)° and the *trans* angles range from 172.60 (5)° to 174.9 (6)°.

## Supra­molecular features   

**Figure 2:**
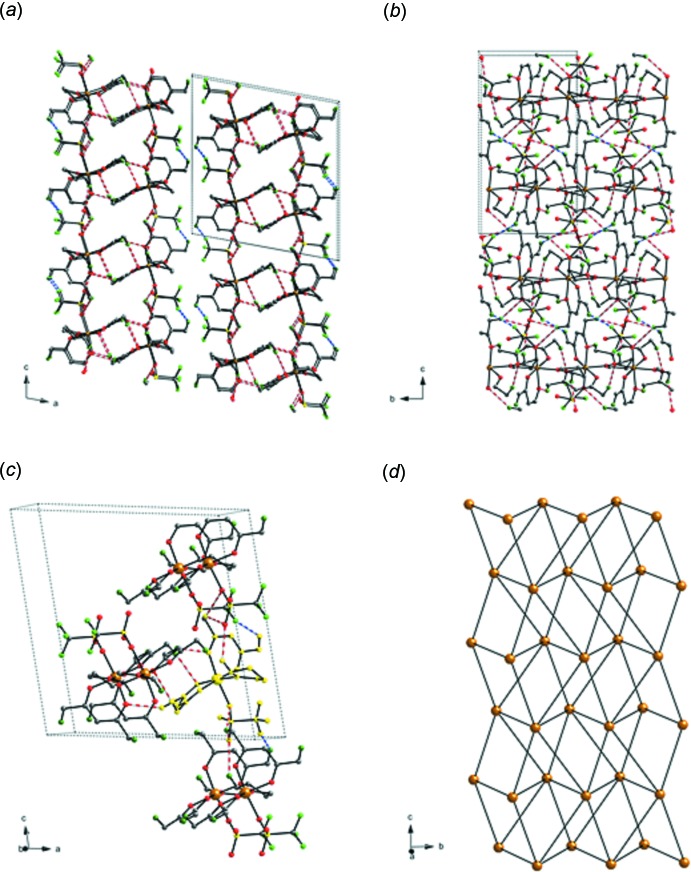
A view of the extended structure of (I) (*a*) along the *b* axis showing two neighboring layers; (*b*) along the *a* axis showing one two-dimensional layer; (*c*) showing the highlighted mol­ecule connecting to six neighboring mol­ecules; and (*d*) reduced to a ball-and-stick representation, orange balls represent one mol­ecule of (I) connecting to six neighbors. H⋯O inter­actions are shown as red dashed lines, H⋯F inter­actions are shown as blue dashed lines. Only the major component of the disordered triflate is shown, and all H atoms except those that participate in the inter­actions are omitted in parts (*a*)–(*c*).

There are no significant supra­molecular features to discuss in the extended structure of (I). There are weak C—H⋯O and C—H⋯F inter­molecular hydrogen-bonding inter­actions resulting in the formation of two-dimensional layers parallel to the (100) plane (Fig. 2 ▸ *a*,*b*). A series of C_meth­yl_—H⋯O_acac_, C_meth­yl_—H⋯O_triflate_, C_meth­yl_—H⋯F_triflate_, C_THF_—H⋯O_triflate_, and C_THF_—H⋯F_triflate_ inter­actions make up the layers, the details of these inter­actions are presented in Table 2 ▸. Each mol­ecule connects to six neighboring mol­ecules through various combinations of these inter­actions, Fig. 2 ▸ *c*,*d*.

## Database survey   

Only one other mononuclear bis­(acetyl­acetonato)iron(III) complex has been characterized crystallographically, [Fe(acac)_2_Cl] (Lindley & Smith, 1970 ▸). This complex comprises a five-coordinate iron(III) atom in a square-pyramidal geometry. The Fe—O distance reported is 1.95 (1) Å, which is comparable to the average Fe—O_acac_ distance in (I) of 1.9668 Å. A search of the Cambridge Structural Database (Groom & Allen, 2014 ▸) reveals twelve bis­(acetyl­acetonato)iron(III) complexes with a Fe—O_acac_ range of 1.945–2.062 Å.

A survey of the database for similar complexes with other transition metals yields one mononuclear bis­(acac)-triflate complex, [Os(acac)_2_(C_6_H_5_)(OTf)] (Young *et al.*, 2011 ▸). There is also only one mononuclear bis­(acac)-THF complex, [V(acac)_2_(Mes)(THF)] (Mes = mesityl; Imhof & Seidel, 2006 ▸). There are six bis­(acac)-bis­(THF) complexes; three mononuclear (Baisch & Poli, 2008 ▸; Döring *et al.*, 1992 ▸; Langer *et al.*, 2007 ▸), two dinuclear (Baisch & Poli, 2008 ▸; Döring *et al.*, 1992 ▸) and one heterometallic tetra­nuclear (Döring *et al.*, 2006 ▸).

## Synthesis and crystallization   

Triflic acid (251 µL, 0.24 g, 1 equiv) was added to a solution of [Fe(acac)_3_] (1 g, 2.83 mmol, 1 equiv) in dry THF (5 mL). The resulting purple–red solution was stirred at room temperature for 1 h. The reaction mixture was then concentrated under vacuum to a volume of approximately 2 mL, and 20 mL of pentane was added. A dark purple–red microcrystalline solid precipitated. The mixture was filtered through a glass-frit and the microcrystalline solid was dried under vacuum (1.25 g, 2.63 mmol, 93%). Crystals suitable for X-ray diffraction were grown by slow diffusion of pentane into a THF solution of the purple–red solid. CH analysis calculated for C_15_H_22_F_3_FeO_8_S (MW: 475.235): C 37.91%; H 4.67%. Found: C 37.69%; H, 4.45%.

## Refinement   

Crystal data, data collection and structure refinement details are summarized in Table 3 ▸. A structural model consisting of the target mol­ecule was developed. The triflate ion is disordered over two positions, with refined site-occupancies of 0.622 (16) and 0.378 (16). The equivalent Fe—O, O—S, S—C, and C—F distances were restrained to be similar (s.u. = 0.01 Å). The disordered atoms were restrained to behave relatively isotropically. Similar displacement amplitudes were imposed on disordered sites overlapping by less than the sum of van der Waals radii. Methyl H atom positions were optimized by rotation about *R*—C bonds with idealized C—H, *R*⋯H and H⋯H distances and included as riding idealized contributors [C—H_meth­yl_ = 0.98 Å with *U*_iso_ = 1.5*U*_eq_(C)]. Remaining H atoms were also included as riding idealized contributors [C—H_methyl­ene_= 0.99 Å and C—H_aromatic_ = 0.95 Å, both with *U*_iso_ = 1.2*U*_eq_(C)].

## Supplementary Material

Crystal structure: contains datablock(s) I. DOI: 10.1107/S2056989015016849/zs2341sup1.cif

Structure factors: contains datablock(s) I. DOI: 10.1107/S2056989015016849/zs2341Isup2.hkl

Supporting information file. DOI: 10.1107/S2056989015016849/zs2341Isup3.tif

CCDC reference: 1423096

Additional supporting information:  crystallographic information; 3D view; checkCIF report

## Figures and Tables

**Table 1:** Selected bond lengths ()

Fe1O1	1.9517(11)	Fe1O8	2.0781(11)
Fe1O4	1.9651(11)	Fe1O5	2.063(4)
Fe1O3	1.9742(11)	Fe1O5*B*	2.066(6)
Fe1O2	1.9762(11)		

**Table 2:** Hydrogen-bond geometry (, )

*D*H*A*	*D*H	H*A*	*D* *A*	*D*H*A*
C1H1*B*O3^i^	0.98	2.53	3.370(2)	144
C5H5*A*O1^ii^	0.98	2.60	3.539(2)	161
C5H5*B*O6*B* ^iii^	0.98	2.56	3.471(12)	154
C6H6*A*O5^iv^	0.98	2.63	3.429(9)	138
C6H6*A*O6^iv^	0.98	2.58	3.436(10)	145
C10H10*B*F3^v^	0.98	2.56	3.214(6)	124
C12H12*A*O6^iv^	0.99	2.51	3.321(7)	138
C12H12*A*O6*B* ^iv^	0.99	2.60	3.423(11)	140
C14H14*A*O7*B* ^vi^	0.99	2.63	3.411(10)	136
C14H14*B*F2*B* ^vii^	0.99	2.50	3.316(6)	139

Symmetry codes: (i) 
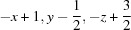
; (ii) 
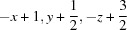
; (iii) 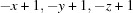; (iv) 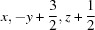; (v) 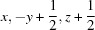; (vi) 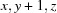; (vii) 
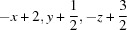
.

**Table 3:** Experimental details

Crystal data	
Chemical formula	[Fe(CF_3_O_3_S)(C_5_H_7_O_2_)_2_(C_4_H_8_O)]
*M*_r_	475.23
Crystal system, space group	Monoclinic, *P*2_1_/*c*
Temperature (K)	103
*a*, *b*, *c* ()	15.0118(8), 8.4523(4), 15.9842(9)
()	100.451(2)
*V* (^3^)	1994.50(18)
*Z*	4
Radiation type	Mo *K*
(mm^1^)	0.93
Crystal size (mm)	0.77 0.09 0.08

Data collection	
Diffractometer	Bruker D8 Venture/Photon 100
Absorption correction	Integration (*SADABS*; Bruker, 2013 ▸)
*T*_min_, *T*_max_	0.720, 0.955
No. of measured, independent and observed [*I* > 2(*I*)] reflections	48981, 4417, 3800
*R*_int_	0.058
(sin /)_max_ (^1^)	0.642

Refinement	
*R*[*F* ^2^ > 2(*F* ^2^)], *wR*(*F* ^2^), *S*	0.025, 0.066, 1.06
No. of reflections	4417
No. of parameters	330
No. of restraints	344
H-atom treatment	H-atom parameters constrained
_max_, _min_ (e ^3^)	0.29, 0.38

Computer programs: *APEX2*, *SAINT*, *XPREP*, *SADABS*, *TWINABS* and *XCIF* (Bruker, 2013 ▸), *SHELXS97* and *SHELXTL* (Sheldrick, 2008 ▸), *SHELXL2013* (Sheldrick, 2015 ▸), *CrystalMaker* (CrystalMaker, 1994 ▸) and *publCIF* (Westrip, 2010 ▸).

## supplementary crystallographic information

### Crystal data

**Table:**

[Fe(CF_3_O_3_S)(C_5_H_7_O_2_)_2_(C_4_H_8_O)]	*F*(000) = 980
*M**_r_* = 475.23	*D*_x_ = 1.583 Mg m^−^^3^
Monoclinic, *P*2_1_/*c*	Mo *K*α radiation, λ = 0.71073 Å
*a* = 15.0118 (8) Å	Cell parameters from 9821 reflections
*b* = 8.4523 (4) Å	θ = 2.6–27.1°
*c* = 15.9842 (9) Å	µ = 0.93 mm^−^^1^
β = 100.451 (2)°	*T* = 103 K
*V* = 1994.50 (18) Å^3^	Needle, red
*Z* = 4	0.77 × 0.09 × 0.08 mm

### Data collection

**Table:**

Bruker D8 Venture/Photon 100 diffractometer	4417 independent reflections
Radiation source: microfocus sealed tube	3800 reflections with *I* > 2σ(*I*)
Multilayer mirrors monochromator	*R*_int_ = 0.058
profile data from φ and ω scans	θ_max_ = 27.2°, θ_min_ = 2.6°
Absorption correction: integration (*SADABS*; Bruker, 2013)	*h* = −19→19
*T*_min_ = 0.720, *T*_max_ = 0.955	*k* = −10→10
48981 measured reflections	*l* = −20→20

### Refinement

**Table:**

Refinement on *F*^2^	344 restraints
Least-squares matrix: full	Hydrogen site location: inferred from neighbouring sites
*R*[*F*^2^ > 2σ(*F*^2^)] = 0.025	H-atom parameters constrained
*wR*(*F*^2^) = 0.066	*w* = 1/[σ^2^(*F*_o_^2^) + (0.030*P*)^2^ + 1.245*P*] where *P* = (*F*_o_^2^ + 2*F*_c_^2^)/3
*S* = 1.06	(Δ/σ)_max_ = 0.001
4417 reflections	Δρ_max_ = 0.29 e Å^−^^3^
330 parameters	Δρ_min_ = −0.38 e Å^−^^3^

### Special details

**Table:**

Experimental. One distinct cell was identified using *APEX2* (Bruker, 2013). Six frame series were integrated and filtered for statistical outliers using *SAINT* (Bruker, 2013) then corrected for absorption by integration using *SAINT*/*SADABS* (Bruker, 2013) to sort, merge, and scale the combined data. No decay correction was applied.
Geometry. All e.s.d.'s (except the e.s.d. in the dihedral angle between two l.s. planes) are estimated using the full covariance matrix. The cell e.s.d.'s are taken into account individually in the estimation of e.s.d.'s in distances, angles and torsion angles; correlations between e.s.d.'s in cell parameters are only used when they are defined by crystal symmetry. An approximate (isotropic) treatment of cell e.s.d.'s is used for estimating e.s.d.'s involving l.s. planes.
Refinement. Structure was phased by direct methods (Sheldrick, 2008). Systematic conditions suggested the unambiguous space group. The space group choice was confirmed by successful convergence of the full-matrix least-squares refinement on *F*^2^. The final map had no significant features. A final analysis of variance between observed and calculated structure factors showed little dependence on amplitude or resolution.

### Fractional atomic coordinates and isotropic or equivalent isotropic displacement parameters (Å^2^)

**Table:**

	*x*	*y*	*z*	*U*_iso_*/*U*_eq_	Occ. (<1)
Fe1	0.70581 (2)	0.58797 (3)	0.74733 (2)	0.01158 (7)	
O1	0.63555 (7)	0.39298 (13)	0.72713 (7)	0.0148 (2)	
O2	0.59623 (7)	0.70589 (13)	0.69469 (7)	0.0149 (2)	
C1	0.53914 (11)	0.19970 (19)	0.65236 (11)	0.0190 (3)	
H1A	0.5792	0.1539	0.6167	0.028*	
H1B	0.4762	0.1933	0.6225	0.028*	
H1C	0.5457	0.1409	0.7060	0.028*	
C2	0.56412 (10)	0.36918 (19)	0.67064 (10)	0.0142 (3)	
C3	0.51150 (11)	0.48966 (19)	0.62755 (10)	0.0161 (3)	
H3	0.4612	0.4605	0.5852	0.019*	
C4	0.52805 (10)	0.65102 (19)	0.64266 (9)	0.0139 (3)	
C5	0.46288 (11)	0.7717 (2)	0.59819 (11)	0.0189 (3)	
H5A	0.4358	0.8302	0.6403	0.028*	
H5B	0.4150	0.7183	0.5583	0.028*	
H5C	0.4952	0.8455	0.5670	0.028*	
O3	0.68185 (7)	0.60779 (13)	0.86430 (7)	0.0160 (2)	
O4	0.82353 (7)	0.49312 (13)	0.79526 (7)	0.0146 (2)	
C6	0.69073 (12)	0.5923 (2)	1.01300 (10)	0.0207 (4)	
H6A	0.6823	0.7058	1.0215	0.031*	
H6B	0.7315	0.5487	1.0625	0.031*	
H6C	0.6320	0.5386	1.0062	0.031*	
C7	0.73100 (11)	0.56757 (18)	0.93464 (10)	0.0145 (3)	
C8	0.81768 (11)	0.5026 (2)	0.94177 (11)	0.0205 (4)	
H8	0.8498	0.4788	0.9972	0.025*	
C9	0.85978 (11)	0.47054 (18)	0.87341 (10)	0.0143 (3)	
C10	0.95457 (11)	0.4060 (2)	0.88703 (11)	0.0211 (4)	
H10A	0.9567	0.3141	0.8501	0.032*	
H10B	0.9730	0.3741	0.9466	0.032*	
H10C	0.9959	0.4878	0.8733	0.032*	
S1	0.7575 (2)	0.4546 (5)	0.5659 (2)	0.0133 (7)	0.622 (16)
O5	0.7471 (8)	0.5722 (10)	0.6313 (4)	0.0148 (14)	0.622 (16)
O6	0.7263 (6)	0.5123 (11)	0.4815 (4)	0.0255 (14)	0.622 (16)
O7	0.7340 (4)	0.2959 (7)	0.5840 (5)	0.0218 (11)	0.622 (16)
C11	0.8807 (3)	0.4490 (8)	0.5760 (4)	0.0251 (11)	0.622 (16)
F1	0.9134 (4)	0.5931 (7)	0.5691 (5)	0.0487 (14)	0.622 (16)
F2	0.9202 (3)	0.3903 (10)	0.6507 (2)	0.0451 (12)	0.622 (16)
F3	0.9047 (7)	0.3580 (11)	0.5158 (6)	0.0374 (15)	0.622 (16)
S1B	0.7611 (4)	0.4453 (9)	0.5676 (3)	0.0166 (12)	0.378 (16)
O5B	0.7429 (13)	0.5656 (16)	0.6296 (7)	0.014 (2)	0.378 (16)
O6B	0.7140 (9)	0.4806 (17)	0.4836 (6)	0.0209 (19)	0.378 (16)
O7B	0.7585 (8)	0.2869 (11)	0.5971 (9)	0.027 (2)	0.378 (16)
C11B	0.8794 (5)	0.4866 (12)	0.5638 (6)	0.0249 (18)	0.378 (16)
F1B	0.8914 (6)	0.6304 (9)	0.5356 (7)	0.0424 (16)	0.378 (16)
F2B	0.9310 (4)	0.4709 (16)	0.6402 (4)	0.047 (2)	0.378 (16)
F3B	0.9104 (11)	0.3852 (18)	0.5118 (9)	0.037 (2)	0.378 (16)
O8	0.76794 (8)	0.80858 (13)	0.75681 (7)	0.0171 (2)	
C12	0.72268 (12)	0.9548 (2)	0.77383 (13)	0.0237 (4)	
H12A	0.6923	0.9419	0.8235	0.028*	
H12B	0.6768	0.9859	0.7239	0.028*	
C13	0.79668 (13)	1.0771 (2)	0.79186 (12)	0.0252 (4)	
H13A	0.8229	1.0812	0.8533	0.030*	
H13B	0.7738	1.1834	0.7728	0.030*	
C14	0.86588 (12)	1.0189 (2)	0.74034 (12)	0.0238 (4)	
H14A	0.8492	1.0511	0.6800	0.029*	
H14B	0.9272	1.0593	0.7639	0.029*	
C15	0.86120 (12)	0.8415 (2)	0.74971 (14)	0.0291 (4)	
H15A	0.8771	0.7876	0.6994	0.035*	
H15B	0.9032	0.8055	0.8012	0.035*	

### Atomic displacement parameters (Å^2^)

**Table:**

	*U*^11^	*U*^22^	*U*^33^	*U*^12^	*U*^13^	*U*^23^
Fe1	0.00926 (11)	0.01369 (11)	0.01071 (11)	0.00065 (8)	−0.00107 (8)	−0.00028 (8)
O1	0.0115 (5)	0.0167 (5)	0.0150 (5)	−0.0007 (4)	−0.0010 (4)	0.0020 (4)
O2	0.0116 (5)	0.0162 (5)	0.0152 (6)	0.0010 (4)	−0.0023 (4)	−0.0011 (4)
C1	0.0140 (8)	0.0176 (8)	0.0242 (9)	−0.0032 (6)	0.0002 (7)	−0.0022 (7)
C2	0.0111 (7)	0.0194 (8)	0.0129 (7)	−0.0026 (6)	0.0044 (6)	−0.0020 (6)
C3	0.0118 (8)	0.0206 (8)	0.0142 (8)	−0.0003 (6)	−0.0021 (6)	−0.0024 (6)
C4	0.0103 (7)	0.0216 (8)	0.0098 (7)	0.0018 (6)	0.0019 (6)	0.0001 (6)
C5	0.0155 (8)	0.0207 (8)	0.0182 (8)	0.0039 (6)	−0.0027 (7)	0.0004 (6)
O3	0.0127 (5)	0.0222 (6)	0.0123 (5)	0.0033 (5)	−0.0002 (4)	0.0000 (4)
O4	0.0117 (5)	0.0179 (5)	0.0132 (5)	0.0025 (4)	−0.0006 (4)	−0.0004 (4)
C6	0.0208 (9)	0.0269 (9)	0.0142 (8)	0.0013 (7)	0.0031 (7)	−0.0014 (7)
C7	0.0154 (8)	0.0140 (7)	0.0133 (7)	−0.0024 (6)	0.0002 (6)	−0.0011 (6)
C8	0.0161 (8)	0.0308 (9)	0.0126 (8)	0.0047 (7)	−0.0025 (6)	0.0030 (7)
C9	0.0118 (8)	0.0116 (7)	0.0178 (8)	−0.0010 (6)	−0.0017 (6)	0.0015 (6)
C10	0.0140 (8)	0.0265 (9)	0.0211 (8)	0.0050 (7)	−0.0013 (7)	0.0018 (7)
S1	0.0083 (9)	0.0132 (9)	0.0184 (12)	−0.0024 (7)	0.0027 (7)	−0.0013 (7)
O5	0.017 (2)	0.015 (2)	0.012 (2)	−0.0008 (19)	0.0022 (19)	−0.0018 (19)
O6	0.031 (3)	0.034 (3)	0.0109 (15)	0.002 (2)	0.0008 (14)	−0.0019 (14)
O7	0.019 (2)	0.0163 (17)	0.032 (3)	−0.0012 (15)	0.0075 (19)	−0.0042 (13)
C11	0.0187 (18)	0.028 (2)	0.030 (2)	−0.0036 (14)	0.0092 (15)	−0.0096 (16)
F1	0.032 (2)	0.040 (2)	0.081 (3)	−0.0199 (16)	0.030 (2)	−0.021 (2)
F2	0.0209 (13)	0.076 (3)	0.0348 (13)	0.0163 (16)	−0.0044 (10)	−0.0075 (17)
F3	0.025 (2)	0.047 (3)	0.043 (2)	0.0016 (19)	0.0147 (16)	−0.0223 (19)
S1B	0.023 (2)	0.0207 (19)	0.0045 (16)	0.0033 (12)	−0.0006 (12)	−0.0060 (12)
O5B	0.016 (4)	0.013 (4)	0.014 (4)	0.000 (3)	0.003 (3)	−0.002 (3)
O6B	0.019 (3)	0.030 (4)	0.012 (3)	−0.009 (3)	−0.0012 (19)	−0.003 (2)
O7B	0.036 (5)	0.016 (2)	0.029 (4)	0.011 (3)	0.010 (4)	0.004 (2)
C11B	0.018 (3)	0.034 (4)	0.022 (3)	0.000 (3)	0.001 (2)	−0.011 (3)
F1B	0.029 (3)	0.038 (3)	0.065 (4)	−0.017 (2)	0.021 (3)	−0.006 (2)
F2B	0.019 (2)	0.082 (5)	0.036 (3)	0.013 (3)	−0.0121 (17)	−0.020 (3)
F3B	0.022 (3)	0.049 (5)	0.044 (4)	−0.002 (3)	0.015 (3)	−0.023 (3)
O8	0.0116 (5)	0.0136 (5)	0.0260 (6)	0.0004 (4)	0.0029 (5)	−0.0043 (5)
C12	0.0208 (9)	0.0162 (8)	0.0346 (10)	0.0029 (7)	0.0063 (8)	−0.0087 (7)
C13	0.0318 (10)	0.0166 (8)	0.0279 (10)	−0.0043 (7)	0.0075 (8)	−0.0068 (7)
C14	0.0225 (9)	0.0190 (8)	0.0299 (10)	−0.0038 (7)	0.0050 (8)	−0.0013 (7)
C15	0.0131 (9)	0.0202 (9)	0.0541 (13)	−0.0033 (7)	0.0066 (8)	−0.0066 (9)

### Geometric parameters (Å, º)

**Table:**

Fe1—O1	1.9517 (11)	C10—H10A	0.9800
Fe1—O4	1.9651 (11)	C10—H10B	0.9800
Fe1—O3	1.9742 (11)	C10—H10C	0.9800
Fe1—O2	1.9762 (11)	S1—O7	1.430 (4)
Fe1—O8	2.0781 (11)	S1—O6	1.431 (4)
Fe1—O5	2.063 (4)	S1—O5	1.472 (4)
Fe1—O5B	2.066 (6)	S1—C11	1.829 (4)
O1—C2	1.2862 (19)	C11—F1	1.324 (5)
O2—C4	1.2827 (19)	C11—F2	1.329 (5)
C1—C2	1.496 (2)	C11—F3	1.332 (4)
C1—H1A	0.9800	S1B—O7B	1.422 (6)
C1—H1B	0.9800	S1B—O6B	1.431 (6)
C1—H1C	0.9800	S1B—O5B	1.479 (6)
C2—C3	1.392 (2)	S1B—C11B	1.822 (6)
C3—C4	1.399 (2)	C11B—F1B	1.319 (7)
C3—H3	0.9500	C11B—F2B	1.330 (7)
C4—C5	1.500 (2)	C11B—F3B	1.335 (6)
C5—H5A	0.9800	O8—C15	1.452 (2)
C5—H5B	0.9800	O8—C12	1.4596 (19)
C5—H5C	0.9800	C12—C13	1.506 (2)
O3—C7	1.2740 (19)	C12—H12A	0.9900
O4—C9	1.2830 (19)	C12—H12B	0.9900
C6—C7	1.501 (2)	C13—C14	1.520 (3)
C6—H6A	0.9800	C13—H13A	0.9900
C6—H6B	0.9800	C13—H13B	0.9900
C6—H6C	0.9800	C14—C15	1.509 (2)
C7—C8	1.398 (2)	C14—H14A	0.9900
C8—C9	1.385 (2)	C14—H14B	0.9900
C8—H8	0.9500	C15—H15A	0.9900
C9—C10	1.503 (2)	C15—H15B	0.9900
			
O1—Fe1—O4	98.09 (5)	C9—C10—H10A	109.5
O1—Fe1—O3	92.44 (5)	C9—C10—H10B	109.5
O4—Fe1—O3	88.33 (5)	H10A—C10—H10B	109.5
O1—Fe1—O2	88.42 (5)	C9—C10—H10C	109.5
O4—Fe1—O2	172.76 (5)	H10A—C10—H10C	109.5
O3—Fe1—O2	94.58 (5)	H10B—C10—H10C	109.5
O1—Fe1—O5	92.2 (3)	O7—S1—O6	117.4 (4)
O4—Fe1—O5	85.9 (3)	O7—S1—O5	115.3 (4)
O3—Fe1—O5	173.1 (3)	O6—S1—O5	112.4 (4)
O2—Fe1—O5	90.7 (3)	O7—S1—C11	103.9 (3)
O1—Fe1—O5B	89.9 (4)	O6—S1—C11	104.2 (4)
O4—Fe1—O5B	86.9 (5)	O5—S1—C11	101.0 (4)
O3—Fe1—O5B	174.9 (6)	S1—O5—Fe1	140.4 (5)
O2—Fe1—O5B	89.9 (5)	F1—C11—F2	107.9 (4)
O1—Fe1—O8	172.60 (5)	F1—C11—F3	108.5 (5)
O4—Fe1—O8	88.72 (5)	F2—C11—F3	107.4 (5)
O3—Fe1—O8	90.64 (5)	F1—C11—S1	110.6 (3)
O2—Fe1—O8	84.63 (5)	F2—C11—S1	111.6 (4)
O5—Fe1—O8	85.4 (2)	F3—C11—S1	110.7 (5)
O5B—Fe1—O8	87.5 (4)	O7B—S1B—O6B	118.0 (7)
C2—O1—Fe1	127.22 (10)	O7B—S1B—O5B	114.0 (7)
C4—O2—Fe1	126.75 (10)	O6B—S1B—O5B	111.4 (8)
C2—C1—H1A	109.5	O7B—S1B—C11B	106.1 (5)
C2—C1—H1B	109.5	O6B—S1B—C11B	104.3 (6)
H1A—C1—H1B	109.5	O5B—S1B—C11B	101.0 (7)
C2—C1—H1C	109.5	S1B—O5B—Fe1	141.8 (8)
H1A—C1—H1C	109.5	F1B—C11B—F2B	108.2 (6)
H1B—C1—H1C	109.5	F1B—C11B—F3B	107.2 (8)
O1—C2—C3	123.98 (15)	F2B—C11B—F3B	107.3 (9)
O1—C2—C1	115.74 (14)	F1B—C11B—S1B	112.5 (5)
C3—C2—C1	120.28 (14)	F2B—C11B—S1B	111.0 (5)
C2—C3—C4	124.09 (15)	F3B—C11B—S1B	110.5 (8)
C2—C3—H3	118.0	C15—O8—C12	109.89 (12)
C4—C3—H3	118.0	C15—O8—Fe1	126.35 (10)
O2—C4—C3	124.06 (14)	C12—O8—Fe1	123.75 (10)
O2—C4—C5	115.87 (14)	O8—C12—C13	105.35 (14)
C3—C4—C5	120.06 (14)	O8—C12—H12A	110.7
C4—C5—H5A	109.5	C13—C12—H12A	110.7
C4—C5—H5B	109.5	O8—C12—H12B	110.7
H5A—C5—H5B	109.5	C13—C12—H12B	110.7
C4—C5—H5C	109.5	H12A—C12—H12B	108.8
H5A—C5—H5C	109.5	C12—C13—C14	103.12 (14)
H5B—C5—H5C	109.5	C12—C13—H13A	111.1
C7—O3—Fe1	129.59 (10)	C14—C13—H13A	111.1
C9—O4—Fe1	129.27 (10)	C12—C13—H13B	111.1
C7—C6—H6A	109.5	C14—C13—H13B	111.1
C7—C6—H6B	109.5	H13A—C13—H13B	109.1
H6A—C6—H6B	109.5	C15—C14—C13	102.66 (15)
C7—C6—H6C	109.5	C15—C14—H14A	111.2
H6A—C6—H6C	109.5	C13—C14—H14A	111.2
H6B—C6—H6C	109.5	C15—C14—H14B	111.2
O3—C7—C8	123.95 (15)	C13—C14—H14B	111.2
O3—C7—C6	116.28 (14)	H14A—C14—H14B	109.1
C8—C7—C6	119.77 (15)	O8—C15—C14	105.13 (14)
C9—C8—C7	124.32 (15)	O8—C15—H15A	110.7
C9—C8—H8	117.8	C14—C15—H15A	110.7
C7—C8—H8	117.8	O8—C15—H15B	110.7
O4—C9—C8	124.44 (15)	C14—C15—H15B	110.7
O4—C9—C10	114.74 (14)	H15A—C15—H15B	108.8
C8—C9—C10	120.83 (15)		

### Hydrogen-bond geometry (Å, º)

**Table:**

*D*—H···*A*	*D*—H	H···*A*	*D*···*A*	*D*—H···*A*
C1—H1*B*···O3^i^	0.98	2.53	3.370 (2)	144
C5—H5*A*···O1^ii^	0.98	2.60	3.539 (2)	161
C5—H5*B*···O6*B*^iii^	0.98	2.56	3.471 (12)	154
C6—H6*A*···O5^iv^	0.98	2.63	3.429 (9)	138
C6—H6*A*···O6^iv^	0.98	2.58	3.436 (10)	145
C10—H10*B*···F3^v^	0.98	2.56	3.214 (6)	124
C12—H12*A*···O6^iv^	0.99	2.51	3.321 (7)	138
C12—H12*A*···O6*B*^iv^	0.99	2.60	3.423 (11)	140
C14—H14*A*···O7*B*^vi^	0.99	2.63	3.411 (10)	136
C14—H14*B*···F2*B*^vii^	0.99	2.50	3.316 (6)	139

Symmetry codes: (i) −*x*+1, *y*−1/2, −*z*+3/2; (ii) −*x*+1, *y*+1/2, −*z*+3/2; (iii) −*x*+1, −*y*+1, −*z*+1; (iv) *x*, −*y*+3/2, *z*+1/2; (v) *x*, −*y*+1/2, *z*+1/2; (vi) *x*, *y*+1, *z*; (vii) −*x*+2, *y*+1/2, −*z*+3/2.
