# Supplementary figures and images for: Crystal structure of bis­(acetyl­acetonato-κ2 O,O′)(tetra­hydro­furan-κO)(tri­fluoro­methane­sulfonato-κO)iron(III)
Source: Acta Crystallogr E Crystallogr Commun. 2015 Sep 12;71(Pt 10):1165–8. doi: 10.1107/S2056989015016849 (PMC4647377; doi:10.1107/S2056989015016849)

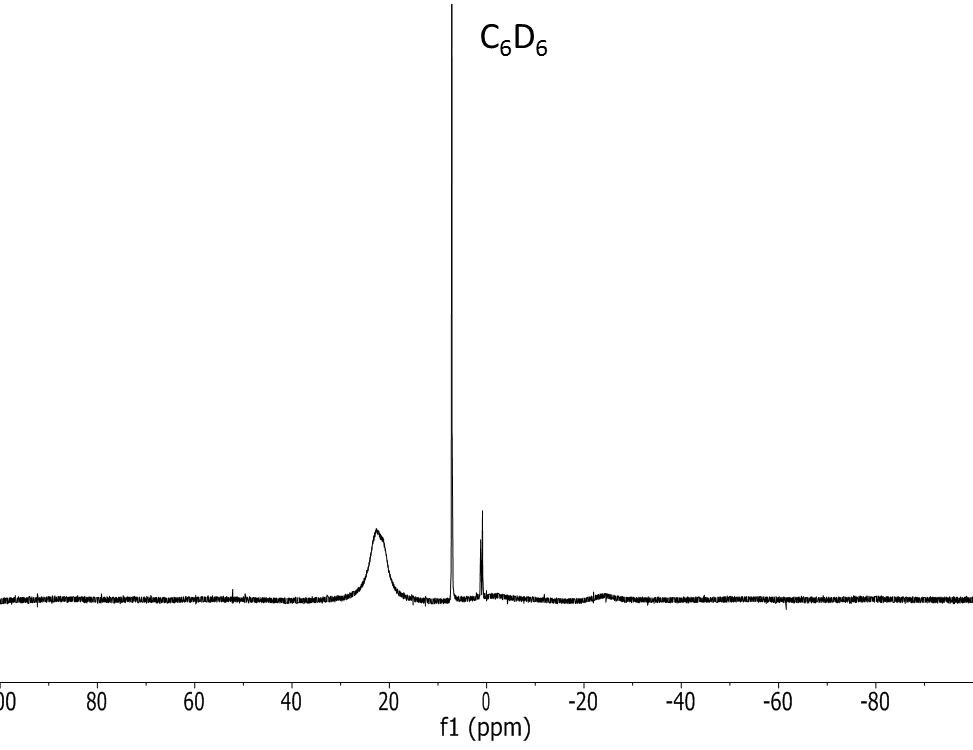

Supplement: Supplementary file 3 [file e-71-01165-Isup3.tif]
